# Dealing with the climate crisis and eco-emotions in psychotherapy – a training for future medical and psychological psychotherapists using standardized patient scenarios

**DOI:** 10.3205/zma001776

**Published:** 2025-09-15

**Authors:** Nadja Gebhardt, Molly Sutcliffe, Jobst-Hendrik Schultz, Hans-Christoph Friedrich, Christoph Nikendei

**Affiliations:** 1 Heidelberg University Hospital, Department of General Internal Medicine and Psychosomatics, Heidelberg, Germany

**Keywords:** eco-emotions, eco-anxiety, competency-based education, standardized patients, psychotherapy training

## Abstract

**Objectives:**

Practitioners and institutions have stated a need for training on how to work with patients who experience various forms of eco-emotions as an addition to existing curricula in psychotherapy training. Even though first suggestions for psychotherapeutic approaches to eco-emotions exist, these have not been translated into training concepts so far.

**Methods:**

The aim of the presented project was to develop and implement a training on dealing with eco-emotions in psychotherapy, based on the framework of competency-based education. A one-day training was developed according to Kern’s cycle. It consisted of i) a lecture, ii) three role-play scenarios with standardized patients, and iii) reflecting group discussions, and was offered three times to a total of n=23 medical and psychological psychotherapists in training at the Heidelberg Institute for Psychotherapy (HIP) in Heidelberg, Germany. The implementation was evaluated regarding feasibility, the educational strategies were evaluated regarding usefulness, and the effects of the training on a gain in competencies were evaluated through self-assessment.

**Results:**

The implementation of the training showed to be feasible. Being an observer during the scenarios and being the acting therapist was ranked to be equally useful for gains in competency. Acceptance for all role play scenarios was high, and participants reported a significant gain in self-assessed competencies.

**Implications:**

Training competencies in working with eco-emotions in psychotherapy is feasible and can be integrated into psychotherapy curricula. Competency-based education as a framework and the use of standardized patients are well suited to train participants in dealing with eco-emotions in psychotherapy.

## Zusammenfassung

**Hintergrund:**

Sowohl Praktiker:innen als auch Institutionen haben einen Bedarf an Training für die Arbeit mit Patient:innen, die verschiedene Formen von Eco-Emotionen erleben, als Ergänzung zu den bestehenden Curricula in der Aus- und Weiterbildung von Psychotherapeut:innen geäußert. Obwohl es erste Vorschläge für psychotherapeutische Ansätze zu Eco-Emotionen gibt, sind diese bisher nicht in Trainingskonzepten umgesetzt worden.

**Methode:**

Das Ziel des vorgestellten Projekts war die Entwicklung und Durchführung eines Trainings zum Umgang mit Eco-Emotionen in der Psychotherapie, das auf dem Rahmen des kompetenzbasierten Trainings beruht. Es wurde ein eintägiges Training nach dem Kern’schen Zyklus entwickelt. Es bestand aus i) einem Vortrag, ii) drei Rollenspielen mit standardisierten Patient:innen und iii) einer Fokusgruppe und wurde dreimal für insgesamt n=23 ärztliche und psychologische Psychotherapeut:innen in Aus- und Weiterbildung am Heidelberger Institut für Psychotherapie (HIP) in Heidelberg, Deutschland, angeboten. Die Implementierung wurde hinsichtlich der Durchführbarkeit evaluiert, die Trainingsstrategien wurden hinsichtlich ihrer Nützlichkeit bewertet und die Auswirkungen des Trainings auf den Kompetenzzuwachs wurden durch Selbsteinschätzung evaluiert.

**Ergebnisse:**

Die Durchführung des Trainings erwies sich als realisierbar. Die Rolle des:der Beobachter:in während der Szenarien und die der handelnden Therapeut:innen wurden als gleichermaßen nützlich für den Kompetenzzuwachs eingestuft. Die Akzeptanz für alle Rollenspielszenarien war hoch, und die Teilnehmer:innen berichteten über einen deutlichen Zuwachs an selbst eingeschätzten Kompetenzen.

**Schlussfolgerung:**

Der Erwerb von Kompetenzen im Umgang mit Eco-Emotionen in der Psychotherapie ist realisierbar und kann in Psychotherapie-Curricula integriert werden. Das kompetenzbasierte Training als Rahmen und der Einsatz von standardisierten Patient:innen sind gut geeignet, um Teilnehmer:innen im Umgang mit Eco-Emotionen in der Psychotherapie zu schulen.

## Introduction

With an accelerating climate crisis, the number of people responding with heightened levels of distress is constantly on the rise [1]. These heightened levels of distress can be summarized under the term eco-emotions. A survey of the literature identified a wide variety of eco-emotions, including threat-related emotions such as fear, anxiety or worry, sadness-related emotions such as grief, hopelessness and helplessness, anger-related emotions such as frustration or indignation, and feelings of guilt or shame [2]. Previous research has mainly focused on fear and sadness and coined terms such as climate anxiety, eco-anxiety or solastalgia [3], [4]. Eco-emotions can express themselves through affective, cognitive, physiological, or behavioral symptoms [5], [6]. They show a negative association with well-being and can negatively affect mental health [7], [8], [9]. Mental health professionals are already reporting an increase in patients mentioning climate-related emotional distress [10]. As younger people (aged 16-25 years) and people who are already experiencing anxious or depressive symptoms in general seem to be especially vulnerable to the experience of impairing levels of eco-emotions [11], [12], [13], the demand for psychiatric and psychotherapeutic support in dealing with eco-emotions will likely grow. Mental health professionals should be well prepared to meet these needs. In recent surveys and interviews, however, mental health professionals report feelings of professional uncertainty and express a wish for additional training on working with eco-emotions [10], [14], [15], [16]. Specifically, 50% of n=414 psychotherapists in Germany reported a lack of information on how to deal with eco-emotions in psychotherapy [16], and out of n=75 mental health professionals in the UK, 37% somewhat agreed and 39% definitely agreed to the statement that they would benefit from specific training [10]. The need for additional training has also been formulated by psychiatric and psychological professional bodies [17], [18], [19], [20].

Eco-emotions display distinct characteristics which might render psychotherapeutic situations challenging. As the concept of eco-emotions is still a novel framework, it lacks a clear definition [3], and is not yet part of psychiatric or psychotherapeutic training. Most research focuses on climate anxiety or eco-anxiety, but the questionnaires developed to assess the construct are based on different definitions and include a wide variety of partially differing symptoms [21]. The lack of international definitions hinders research and results in varying prevalence of eco-distress and different correlations with mental health measures such as depressive or anxiety symptoms [13], [21], [22]. Thus, mental health professionals might experience a lack in knowledge. Moreover, mental health professionals and patients share the experience of living in a world with an accelerating climate crisis. Therefore, mental health professionals might feel the need to reflect and consolidate their therapeutic stance and personal attitudes regarding the climate crisis when eco-emotions become a topic in psychotherapy. 

Finally, the climate crisis is multi-faceted, influencing people at the individual and societal level and creating the wide variety of emotional reactions. While the affective and physiological experience of eco-emotions does not differ from the experience of emotions in general, the accompanying cognitions and behaviors might. For example, people diagnosed with generalized anxiety disorder usually report anxiety related to a variety of topics, and they tend to overestimate the likelihood of something negative happening to them or their significant others [23]. In contrast, anxiety about the increasing and self-accelerating consequences of the climate crisis is based on a valid assessment of the facts, and individuals tend to fear not only for themselves and their loved ones, but also for humanity in general [9]. Thus, established psychotherapeutic approaches have to be adapted to be helpful in the new contexts. Previous research has suggested various approaches to draw upon when dealing with eco-emotions in psychotherapy, e.g. acceptance-commitment-therapy, existential psychotherapy, emotion-, action-, and meaning-focused coping, cognitive restructuring, or containment and transformation of inner ambivalences [24], [25], [26], [27]. Thus, mental health professionals might need assistance in identifying and developing the necessary psychotherapeutic skills to work with eco-emotions in psychotherapy.

The model of competency-based education is well suited to address the above-named challenges when it comes to dealing with eco-emotions. Within this framework, competencies can be understood as the knowledge, skills, and attitudes specific to the task at hand [28]. Competency-based teaching models provide a comprehensive learning environment, wherein knowledge, attitude, and skills interact to encourage the growth of clinical competence [29]. Specifically, lectures have been shown to be suitable for knowledge acquisition, whereas role-plays and standardized patients have been deemed suitable for attitude and skill development [30]. In psychiatry training and assessment, employing standardized patients is already common practice [31]. In psychotherapy training and assessment, however, the potential of standardized patients has only been discovered in recent years [32]. One example is the “DYNAMIK”-curriculum for psychodynamic psychotherapists in training, which uses standardized patients in teaching and assessment of conflict- and structure-oriented intervention strategies [33], [34]. However, it remains unclear so far whether this approach can be transferred onto the training of competencies in dealing with eco-emotions in psychotherapy. 

The research questions guiding our project were:

- How to develop and implement a training addressing the distinctive features of eco-emotions in psychotherapy for future medical and psychological psychotherapists?
- Would participation in such a training result in an improvement in knowledge, attitude, and skills regarding this topic?

## Training development

### Theoretical framework

The training was developed according to the six-step approach formulated by Kern [35]. The six steps are:

1. problem identification and general needs assessment,
2. targeted needs assessment,
3. goals and objectives,
4. educational strategies,
5. implementation,
6. evaluation.

The application of step (1) to (4) in our project is described in the following section. The (5) implementation and (6) evaluation are described in the results section of this manuscript. 

Problem identification and general needs assessment

The demand for psychosocial support in dealing with eco-emotions is growing, thus health care providers should be enabled to meet this demand. However, while there are many theoretical considerations on how to deal with the topic in psychotherapy [24], [25], [27], evidence-based examples for psychosocial interventions are scarce [36], and no guidelines or examples of how to train practitioners in dealing with eco-emotions have been published so far.

### Targeted needs assessment

As outlined in the introduction, practitioners report a professional uncertainty in dealing with eco-emotions in psychotherapy and wish for additional training. Specifically, they wish for more information on the climate crisis and mental health [14]; for guidance on how to approach the topic in a professional and ethically sound way in psychotherapy [15]; and for training on how to adapt general skills, like conveying coping strategies, to the specific context of the climate crisis [10]. 

### Goals and objectives

The overall aim of the training was to achieve a gain in participants’ knowledge, attitudes, and skills regarding eco-emotions in psychotherapy. Specifically, participants should acquire knowledge about the consequences of the climate crisis on mental health, possible forms in which these could become relevant in psychotherapy, and an overview of intervention strategies formulated in the literature so far. They should have the possibility to reflect their own emotional reactions to the climate crisis and how these might interact with the therapeutic situation, as well as their personal attitudes both towards the climate crisis and towards patients reporting climate-change related mental health impairments. Finally, they should be enabled to employ different interventions to address different aspects of the interplay of the climate crisis and mental health. 

### Educational strategies

Knowledge acquisition should be achieved through a lecture on climate crisis, mental health, and psychotherapy. The reflection of one’s attitude and therapeutic stance, as well as the use of different interventions to address eco-emotions in psychotherapy, should be trained via different scenarios with standardized patients and a group discussion. The scenarios were inspired by the research team’s practical experience as psychotherapists, the work of one of the researchers (N.G.) as a counselor for climate activists, and the scientific literature on the nature of eco-emotions and its implications for psychotherapy [24], [25], [27], [36]. The term standardized patient is an umbrella term for both a well-trained person simulating a patient’s symptoms in a standardized way and an actual patient presenting their symptoms in a standardized way [37]. For the training presented here, standardized patients would be recruited from the standardized patient pool of the medical faculty of Heidelberg University, Germany [38], to simulate symptoms in a standardized way. Finally, group discussions can generate new insights and perspectives as a result of the conversational process [39], [40].

## Methods

### Participants

Medical and psychological psychotherapists in training, as well as psychology students who had already gathered clinical experience, were eligible to participate. Participants were recruited via e-mail announcements of the training. While the training was free of charge, it was not part of the mandatory course work for medical and psychological psychotherapists in training. In total, *n*=23 persons participated during three one-day seminars. Group size was *n*=6, *n*=7, and *n*=10. Of these, *n*=19 were psychological psychotherapists in training, *n*=2 medical psychotherapists in training, and *n*=2 psychology students. Participants were *M*=29.8 (*SD*=4.02) years old, *n*=20 (87%) participants identified as female, and *n*=3 (13%) as male. On average, participants had *M*=21.4 (*SD*=17.5) months of clinical experience. The study was approved by the ethics committee of the Medical Faculty of the University of Heidelberg (S-098/2024) and was in line with the Declaration of Helsinki.

### Feasibility of the training

The training would be considered feasible if it could be conducted multiple times without the need to alter the procedure.

### Usefulness of the training modules

The modules of the training were ranked 1-5 regarding their usefulness for a gain in competencies by the participants. The approach was adapted from the evaluation of the “DYNAMIK”- curriculum [34], [41], which is also based on the six-step approach for curriculum development formulated by Kern [35].

### Assessment of the scenarios with the standardized patients

Directly after the scenarios, those were rated by the participants on a six-point Likert scale regarding realism, utility, difficulty, and acceptability. The items assessing realism, usefulness, and difficulty were adapted from a previous study comparing peer role play and standardized patients [42]. The items were “The session with the standardized patient was realistic in terms of the depicted psychological impairments and thoughts about the climate crisis” (realism); “The session with the standardized patient was useful for practicing how to handle psychological impairments related to the climate crisis in a psychotherapeutic context” (utility); and “The session with the standardized patient was difficult” (difficulty). Possible answers ranged from “strongly disagree” to “strongly agree”. An item assessing acceptability was added due to the novelty of eco-emotion-focused scenarios. To this end, we asked participants to give a grade for the scenario, ranging from “unsatisfactory” to “very good”.

### Self-assessed gains in competencies

The six-step approach for curriculum development formulated by Kern [36] is based on the concept of competency-based education [43]. Competencies refer to specific knowledge, attitudes, and skills, with competencies themselves being elements or components of competence [44]. Consequently, participants’ gain in competencies was assessed via three items asking for self-assessed knowledge, attitudes, and skills. The items are based on the corresponding items used in the evaluation of the “DYNAMIK”- curriculum, the development of which is also based on the concept of competency-based education [34], [41]. The items were “I am knowledgeable about psychological impairments related to the climate crisis that are relevant in psychotherapeutic treatment” (knowledge); “I have a reflected attitude towards dealing with psychological impairments related to the climate crisis in psychotherapy” (attitude); and “I feel capable of addressing psychological impairments related to the climate crisis in psychotherapy” (skills). Participants answered on a six-point Likert scale, ranging from “strongly disagree” to “strongly agree”. Competencies were assessed before and after the training, and evaluated with a paired t-test (Bonferonni-corrected for multiple testing).

## Results

### Implementation 

The whole training consisted of three modules and lasted for six hours, the procedure is displayed in table 1 (Tab. 1). After an initial lecture, participants were split into groups of 2-3 people and presented with three scenarios with standardized patients. Participants took turns being the therapist or observing the scenario. After going through all three scenarios in small groups of two or three, a group discussion was conducted with the whole group (10≤*n*≥6).

### Lecture

The lecture aimed to give an overview of the current knowledge on eco-emotions, its relationship with psychological impairments, and postulated treatment approaches in psychotherapy and counseling. Specifically, different emotional reactions to the climate crisis and their implications for mental health were presented, and the possibly enhanced vulnerability of persons with pre-existing mental health impairments [45], [46] was highlighted. Afterwards, common factors of successful psychotherapy, as well as the scientific literature regarding different treatment approaches for treating eco-emotions in psychotherapy, were reviewed.

### Standardized patient scenarios

The three scenarios were designed to cover a broad range of emotional reactions to the climate crisis and to address different interventional skills. Table 2 (Tab. 2) gives an overview of the three scenarios. The detailed scenarios handed out to the standardized patients to practice their roles are provided as attachment 1 . Participants received a shortened version without the information which topic the patient would discuss today. 

### Group discussions

To reflect upon their own emotional reactions to the climate crisis in general and to the scenarios with the standardized patients in particular, a group discussion was conducted. It addressed both the process of acquiring new competencies and the role of common factors of successful psychotherapy. Common factors of successful psychotherapy which were specifically addressed were congruence, empathy, and the therapeutic relationship [34]. 

### Evaluation

#### Feasibility of the training

The training was conducted multiple times without the need to alter the procedure. Thus, it was considered feasible.

#### Usefulness of the training modules

Descriptively, being an observer during the scenarios was ranked to be equally important for gains in competency (*M*=2.57, *SD*=1.25) as being the therapist (*M*=2.57, *SD*=1.57), followed by the lecture (*M*=3.29, *SD*=1.38), the standardized patients’ feedback (*M*=3.48, *SD*=1.60), and the peers’ feedback (*M*=3.52, *SD*=1.25).

#### Assessment of the scenarios with the standardized patients

Overall, ratings for all scenarios were favorable. Scenario 1 (angry climate activist) was perceived to be the most realistic, useful, and acceptable, and scenario 3 (grieving mother) was perceived to be the most difficult. All ratings are displayed in table 3 (Tab. 3).

#### Self-assessed gains in competencies

Participants reported significant gains in knowledge (*t*=8.51, *p*<0.001, *Cohen’s D*=1.81), attitudes (*t*=4.50, *p*<0.001, *Cohen’s D*=0.96), and skills (*t*=7.28, *p*<0.001, *Cohen’s D*=1.55).

## Discussion

Based on the existing literature, we developed a training for future medical and psychological psychotherapists on how to deal with eco-emotions in psychotherapy as a missing resource to adapt the health care system to the new challenges which are arising through the climate crisis. Guided by the six-step approach of curriculum development, we consecutively defined learning objectives and the fitting educational strategies. The result was a one-day training, consisting of a lecture, three scenarios with standardized patients, and a group discussion. The implemented training showed to be feasible, as it was successfully conducted three times with three different groups without alterations to its procedure or content. Moreover, participants reported a high acceptance for all scenarios with standardized patients, the realism, usefulness, difficulty, and acceptability to be above average. This translated into a self-assessed gain in knowledge, attitudes, and skills. 

Regarding the participants’ rating of the different modules, observing or being the therapists during the scenarios with standardized patients was rated descriptively as most important for gains in competency by the participants. This is in line with a previous integration of standardized patients into psychotherapy training and assessment [41], and highlights the possible benefits of a more frequent integration of this educational strategy into psychotherapy training. Interestingly, Scenario 2 (ambivalent engineer) was perceived as contributing less than the other two scenarios to a gain in competencies. In this scenario, the patient discusses his conflict with his girlfriend about taking a flight to go on holiday. The patient argues that such a flight would produce unnecessary greenhouse gas emissions, she surmises he wants to avoid a lengthy trip as work is more important to him than their relationship. During the group discussion, participants reported that often in this scenario, they had concentrated on the relationship, and less on climate change-related aspects. In part, this had been intended by the research team, as our aim was to design realistic scenarios that could happen in a similar fashion during regular psychotherapies. For training purposes, however, it seems that a clear focus on the topic at hand is more favorable to enable participants to develop topic-specific competencies. However, the climate crisis will rarely be the sole topic in psychotherapy, thus offering only scenarios in which patients focus completely on it might be unrealistic. Overall, therefore, there are arguments both for replacing the scenario and for retaining it.

The training presented in this study translates existing theoretical considerations on how to address eco-emotions in psychotherapy into practical, experiential learning. While the existing literature outlines a range of psychotherapeutic approaches – such as acceptance-commitment therapy, existential psychotherapy, and meaning-focused coping – it often stops short of offering concrete guidance on how to operationalize these frameworks in clinical practice. By offering the participants an overview of the possible psychotherapeutic approaches, as well as the possibility to practice their usage in different scenarios, the training bridges conceptual understanding and therapeutic action. This practical implementation allows for a deeper integration of theory into the therapeutic repertoire, supporting medical and psychological psychotherapists in training in confidently addressing eco-emotions in psychotherapy. Furthermore, our approach underlines the potential of extending the use of standardized patients in psychotherapeutic training to the area of eco-emotions.

### Limitations

As the evaluation of the training was based on participants’ subjective evaluation, an objective assessment of a possible gain in competencies and its transfer to psychotherapy sessions with real patients is missing. This could be achieved through the blinded assessment of video-taped training and psychotherapy sessions of participants before and after the training, or through written knowledge tests. Likewise, a comparison with other trainings on eco-emotions in psychotherapy adapting other psychotherapeutic approaches would have to be undertaken by future research projects [47]. Moreover, the theory of cognitive dissonance predicts that individuals would perceive an activity such as our training more favorably because of the time and effort they invested, as they need a justification for their investment [48]. An objective assessment would help to ascertain whether participants’ favorable assessment of the training was grounded in this effect or rather in the actual quality of the training. 

Furthermore, participants had limited practical clinical experience with which to compare the quality of the scenarios in our training. This was partly due to the novelty of the phenomenon, partly due to the lack of practical experience in our participants. Moreover, it remains unclear whether the eco-emotions covered in our scenarios are sufficient to prepare participants for working with eco-emotions in psychotherapy. Our selection was guided by a taxonomy of eco-emotions based on the existing literature [2], thus we are confident that we included the most prominent ones. However, future research would have to determine an optimal training intensity and whether all of the different eco-emotions would have to be addressed. Finally, the training has so far only been implemented in one institution, thus transferability to other institutions has yet to be ascertained by future research. The use of standardized patients renders the implementation more challenging, as this requires an additional pool of personnel and an infrastructure to provide a sufficient amount of training for the standardized patients in fulfilling their roles. A possible solution might be to use peer role plays instead, e.g. by having the trainees take turns imitating the patient. Such an approach would offer the possibility to foster empathy for patients’ perspectives by simulating them [49].Moreover, peer role play and standardized patients have been shown to be comparably valuable in medical training [50].

## Conclusion

The training on eco-emotions in psychotherapy for medical and psychological psychotherapists described here represents one approach to implementation into psychotherapy curricula. The training procedure has been shown to be feasible, and the trainings’ contents were rated to be acceptable and useful for a gain in competencies by the participants. Moreover, the novel aspect of standardized patients was evaluated favorably by the participants and could be extended to other trainings in the future. Subsequent research has to evaluate whether the subjective gains in competency reported by the participants will also translate into better outcomes in patient symptomology. 

## Authors’ ORCIDs

- Nadja Gebhardt: [0000-0001-9353-5394]
- Jobst-Hendrik Schultz: [0000-0001-9433-3970]
- Hans-Christoph Friederich: [0000-0003-4344-8959]
- Christoph Nikendei: [0000-0003-2839-178X]

## Competing interests

The authors declare that they have no competing interests.

## Supplementary Material

Patient scenarios

## Figures and Tables

**Table 1:**
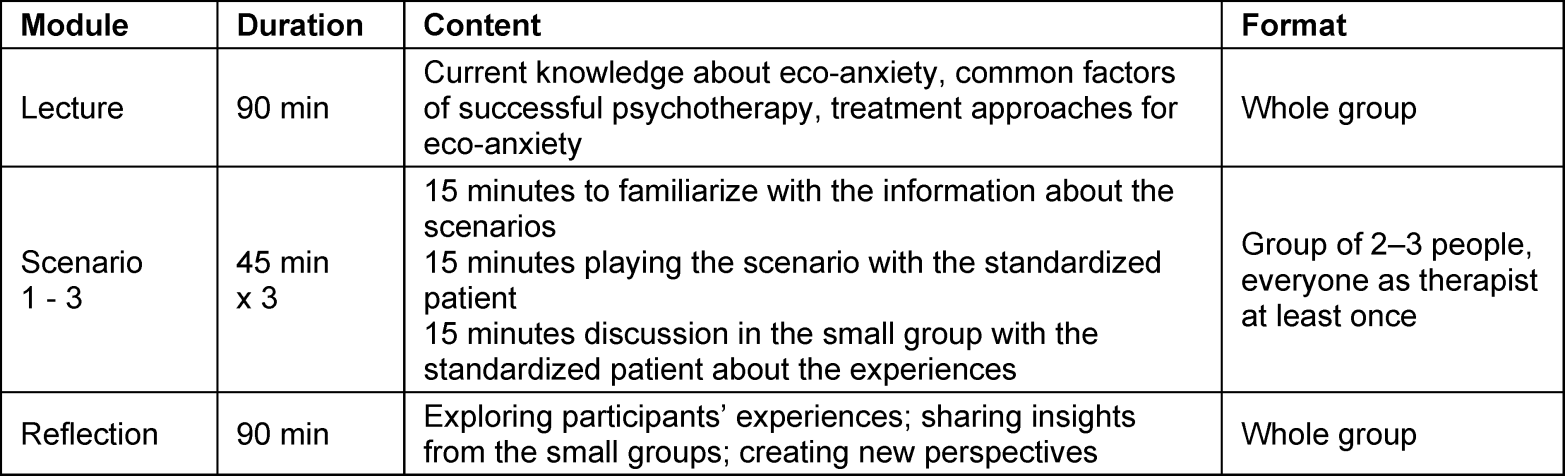
Training procedure

**Table 2:**
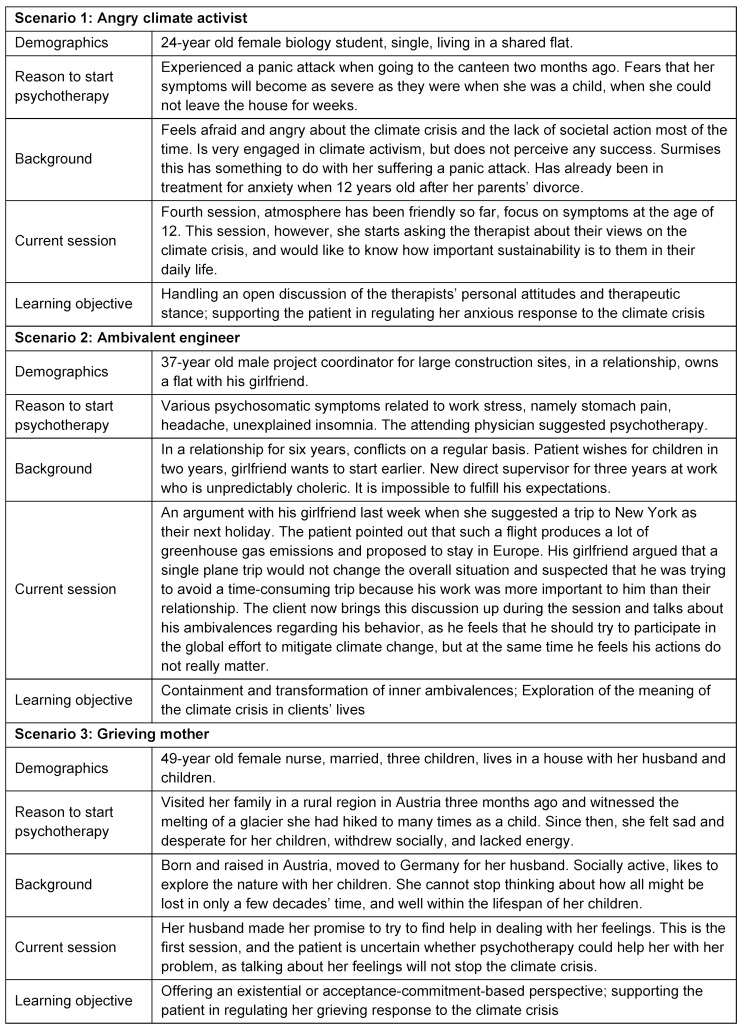
Overview of the scenarios participants were confronted with by the standardized patients

**Table 3:**
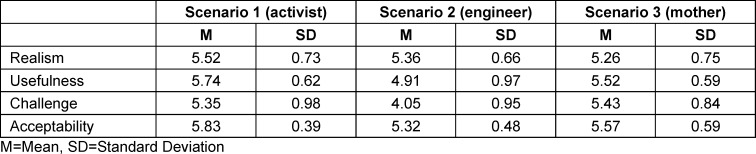
Assessment by the participants of realism, usefulness, challenge, and acceptability per scenario played out with the standardized patients
