# Supplementary material for: Dealing with the climate crisis and eco-emotions in psychotherapy – a training for future medical and psychological psychotherapists using standardized patient scenarios
Source: GMS J Med Educ. 2025 Sep 15;42(4):Doc52. doi: 10.3205/zma001776 (PMC12527395; doi:10.3205/zma001776)
Supplement: Patient scenarios [file JME-42-52-s-001.pdf]

Attachment 1: Patient scenarios

Case history (I) seminar on psychological stress caused by the climate crisis

Ms. Steiner, 27 years old

Ms. S., single, studying for a master's degree in biology in Heidelberg, lives in a shared flat.

Complaints

Since the beginning of your studies, you have been working intensively on the effects of climate change on the planet and are very concerned about the consequences. So far, you have been able to discuss this with your friends, but for the past few months this has no longer been enough. Your thoughts are constantly revolving around this topic, you are constantly afraid of the future, you find it difficult to make the simplest decisions, such as choosing what to eat for lunch, if you can't estimate which dish caused more emissions during production. You are particularly concerned about microplastics in the oceans, as you are aware of how harmful they are for the entire planet, especially in light of your studies. In the evening, you lie awake for hours and dystopian scenes play out in your mind's eye before you fall asleep. During the day, you spend many hours worrying and thinking about what you could do to prevent it, because you are convinced that it depends on each individual. At the same time, you feel a great sense of anger when you think about how many people in Germany and around the world don't seem to care that they are destroying their own livelihoods. You simply cannot believe that there are people who can ignore this. You have been eating too little for months, you simply have no appetite. When you were 12 years old, your parents separated. The following year, you developed an anxiety disorder and were unable to go to school for weeks. You were in outpatient psychotherapy at the time, which helped you a lot. You remember that you talked to the therapist about the situations and thoughts that made you anxious and that things slowly got better. You have not been in treatment since then and did not need it until a few months ago.

Current triggers

Two months ago, you suddenly felt very anxious again when you were in the canteen and you had to leave the building quickly to get your breath back. You believe that this incident is related to the fact that you are afraid all the time because of climate change. You are afraid that the symptoms will become as severe as when you were a child and couldn't leave the house for weeks. You want to prevent that at all costs. In addition, you now have responsible tasks at Scientists for Future and want to be able to continue doing them, you are involved in planning information days and other activities. Since this incident, you no longer go to the canteen, but you remember from your first therapy that avoidance behavior does not reduce your anxiety. When you are extremely angry or anxious, you are also unable

to concentrate on anything else. You would therefore like to find a different way of dealing with these feelings so that you can continue to be fully committed to climate protection.

Life story

Family of origin

You have a younger brother (-10) who is currently doing his A-levels and lives with your mother in Karlsruhe. They remember a wonderful childhood, with lots of trips to child-friendly exhibitions or to the Black Forest. Your father was hardly ever at home during the week, as he had to work all over Germany. When you were 12 years old, your parents separated and your father moved to the Ruhr area shortly afterwards. They only go there at Christmas, and these days are always very stressful for you, as they don't really have anything to say to each other. Your father has a new wife, no more children and lives in a modern apartment. His partner is always nice to you, but you don't like her very much. Your father works as an engineer and earns a good salary, and it was at work that he met his current partner. Conversations often revolve around how you can be successful in life. If you try to talk about climate protection, your father will quickly become angry and won't listen to you. He says the market will sort it out. You have a very close relationship with your mother, you describe her as your best friend. Your mother works part-time in a bakery and supports you in your political activities, but is always worried that you don't take enough time to rest. Since the separation, your mother's financial situation has always been strained, you blame your father for this because he doesn't pay your mother any maintenance, your mother says he is "raking it in". They admire their mother's energy for looking after the children so devotedly all these years and for having a job. Your brother is very musical and plays the piano and flute, which are the only things that interest him at the moment. You also have a very good relationship with him, sometimes you feel like a second mother to him.

Partnerships

You are not currently in a steady relationship and have never been for more than a year. There are always men who interest you, but they usually don't share your political views in their cosequence, and you don't want to be with someone like that. You currently have a friend in Scientists for Future, a chemistry student who you find very attractive. However, his mother has recently passed away and he has no head for romantic relationships at the moment. However, you have decided to give him some time, as you are impressed by his intellect and his uncompromising attitude in discussions about climate protection.

Work / Professional

You spent both your Bachelor's and Master's degrees in Heidelberg, and you spent a semester in Bergen, Norway, during your Bachelor's degree. You chose biology as your subject because it was your best subject at school and you have always felt a connection with nature. In addition, your best friend from

high school also decided to study biology in Heidelberg, and you are still very close friends and also share their views on climate change.

Social situation / hobbies

You live in a 4-bed flat share in Heidelberg and get on very well with 2 of your flatmates, one of whom is very messy, which often leads to conflicts. Overall, however, you find living in a shared flat very nice. Your mother works part-time, so you are entitled to BAföG, and your father also pays you monthly maintenance. Overall, this is enough to cover your running costs; you don't have any major savings. Your circle of friends in Heidelberg consists mainly of fellow students, with whom you also go bouldering together. You also play the violin in a recreational orchestra in Heidelberg. You spend about every other weekend with your mother and still have an intact circle of friends there; you also see your younger brother then. As part of your studies, you have met many people who are very interested in environmental policy issues and have gathered a lot of information on the subject. You also regularly go to demonstrations for climate protection, eat a vegan diet, try to recycle as many things as possible, don't fly and try to produce as little plastic waste as possible.

Psychological findings

You don't pay much attention to your appearance, but cleanliness is very important to you. Their appearance is therefore usually careless and functional, but never dirty. During the sessions, you look your therapist openly in the eye and speak in a quiet but emphatic voice, often quickly. They usually hold themselves upright. They report fears about the future,

climate change, sometimes also their financial situation, and a worsening of their symptoms. Their mood is usually desperate and they often cry without being able to give a reason. They have difficulty falling asleep and are sometimes very hungry, sometimes not hungry at all. They have no thoughts that are tired of life. They are usually tense and agitated and try to counter this by consciously breathing out.

Climate change-related aspects

You have already had many experiences of rejection regarding your attitudes to climate change and are not yet sure what your therapist thinks about this. So far, she has listened to everything with a nod, but you don't know what she herself thinks. At first you thought it wasn't that important for you, but in the last week you've realized that this isn't true. You believe that you need a therapist who has a realistic perspective on world events regarding climate change. You also become quite angry at the thought of the possibility that you might be confiding in someone who doesn't take your fears seriously.

Course of therapy (3 hours so far)

You have been to three appointments with your psychotherapist so far. At the beginning, you had high hopes that she would be able to help you deal with your feelings in a different way. The atmosphere during the first sessions was very friendly. You mainly talked about your symptoms at the age of 12 and your current anxieties. In the last hour, you started to think together about what realistic goals could be for the therapy. By the end of the session, they had agreed that a significant reduction in anxiety symptoms would be their primary therapy goal.

Start of lesson

You have decided to address the therapist's attitude to climate change directly today: what is your therapist's position on this? What is she herself doing to protect the climate? And how does she cope with the fact that we as a society are failing to protect the climate - in other words, how does she cope with her own failure to protect the climate? You are very upset and afraid of crossing a line, but you force yourself to broach the subject. You start the lesson by describing your thoughts:

"I've talked a lot about myself and my views on climate change in the last few sessions. I realize that the therapy is about me and not about you. But I just don't feel comfortable if you only nod noncommittally and I don't even know what you think about it. I also don't think you can help me if you don't understand me on this point."

Please remain silent afterwards and wait for the therapist’s reaction...

Case history (II) seminar on psychological stress caused by the climate crisis

Mr. Benninghaus, 37 years old

Mr. B., 37 years old, project coordinator for large construction sites, in a relationship without children, undergoing treatment for psychosomatic complaints.

Complaints

You started psychotherapy because you often went to the doctor with numerous physical complaints, mostly pain in your back or stomach, but also headaches or insomnia that you could not explain. Your general practitioner finally referred you to her, stating that a stress-related cause of your complaints could not be ruled out. You experience your mood as actually always balanced, you are "simply not the type" for extreme emotions such as anger or joy. You feel that this is particularly important in your relationship with your wife in order to be able to offer her emotional stability. Recently, however, you have noticed that you are more irritable than before, and your partner also says that you are tense and pay less attention to her. You would say that you have always been fine, but your wife has told you that your symptoms have been getting worse for several years.

Current triggers

However, what finally motivated you to start psychotherapy was the observation that you were significantly less productive at work than before. A few weeks ago, after harsh criticism from your boss, you suddenly felt like you were losing your balance and when you returned to your desk, you found it impossible to concentrate. You then went home during your lunch break. That would never have happened to you before, you would have been able to stand it. Even if your wife tells you that you are tense, you don't notice it. The thought that there are processes in your body that you don't notice and that may even lead to symptoms as severe as pain scares you. But you would never admit this. In your self-image, you are efficient and agile and do not show any weaknesses so as not to worry others. Being in control of the situation is important to you.

Life story

Family of origin

You have a younger sister (- 4) and grew up with your married parents in a small village in Baden-Württemberg. You experienced your father as strict but fair, your mother as often overwhelmed by life, then she was unpredictable and often hit you without warning. Although you would never hit your own children, you defend your mother's behavior: she simply didn't know any better. Your parents both come from the village where you lived as a child. After finishing secondary school, your mother completed an apprenticeship as a seamstress, but has not worked since the children were born. Her father graduated

from secondary school and trained as an electrician. He has worked in the same family business since graduating. Overall, you describe your childhood as very harmonious. Your parents never argued. However, raising the children was mainly the mother's job and their father never interfered too much. You did well at secondary school and then transferred to the local grammar school to do your A-levels. You don't remember arguing much with your parents in your youth. You were already a calm and balanced character back then, so there was no reason for conflict. Nowadays, you visit your parents about once a month and also spend holidays such as Christmas and Easter with them. Your girlfriend moved here from northern Germany and usually comes to visit your family.

Partnerships

You have been in a stable partnership for 6 years. You met your girlfriend at work. She works for a large software company where you had a contract to build a new building. Your girlfriend also studied business administration and works in the HR department. You had to coordinate the division of the offices with her, which is how you came into contact. You experience your relationship as very harmonious, you can both live with resolving any disagreements before going to bed. However, it sometimes bothers you that your girlfriend seems to have a problem much more often than you do. However, you would never tell your girlfriend this. You just think she's a bit more emotional and it's your job to provide a calm balance. You are thinking about having children in 1-2 years. As your girlfriend is 5 years younger and would then be 33, you think this is a good time. When you bought your current apartment 3 years ago, you made sure that it also had a children's room, which you currently use as a guest room. Before your current relationship, you were in a steady relationship with a classmate from the age of 17 until the age of 29. She finally ended the relationship because she stayed in your home village and it was clear that you would not be able to pursue your career there, but her ex-partner did not want to move away either. The separation had made them very insecure at the time and they are now happy to be back in a stable partnership. In the two years that you were single, you focused primarily on your career and worked a lot.

Work / Professional

You are currently working as a project coordinator on large construction sites. You chose this job because it pays well, but you don't have to do much physical work yourself and therefore don't have to worry about having health problems at some point. You have been doing this job for 8 years and plan to continue it until further notice. What is causing you problems, however, is your direct superior. He joined the company 3 years ago and is unpredictably choleric. You are very frustrated that you can't seem to please him, no matter how hard you try.

After leaving school, you initially trained as a gas and water fitter and worked in this profession for several years. You moved to Mannheim for this purpose. A colleague of yours quit to study; you found

this idea very appealing, but could not imagine living in such financial insecurity. After some research, you therefore started a distance learning course in business administration, which you successfully completed alongside your full-time job. With this and your practical experience, you were ultimately excellently qualified for the job you now have, as it often involves coordinating theoretical ideas and construction plans with the practical requirements on site. You also find it very enjoyable to plan and coordinate and to be paid for keeping an overview.

Social situation / hobbies

You live in a condominium in Heidelberg and are very proud of what you have achieved. In your youth, you were always extremely active in sports, practiced table tennis and swimming at the same time and never spent much time at home. You had a few friendships, but none of them have lasted to this day, which you explain by the fact that you no longer live in your home village. You gave up sport completely a few years ago, as your job demands too much of you. You don't really have any friends, but your wife is well connected socially and often takes you to events.

Psychological findings

You are always neatly dressed, shirt with jeans. When talking to the therapist, you are polite and try to make the conversation as pleasant as possible. You speak clearly and tend to speak slowly, in a calm voice. Your demeanor is very controlled and you are rarely able to maintain eye contact when you talk about your difficulties. You are very afraid of losing your job or

your partner because of the current conflicts, but you are reluctant to express this because you are ashamed of being so weak. The thought of death also causes you great anxiety because you are worried that you are not living your life to the full. Your mood is usually depressed, even if you say you are "balanced". You have a feeling of inner restlessness and often feel the need to fidget or tap your foot, but you consistently suppress this and try to sit as motionless as possible. You often have phases of severe exhaustion in the evening, in which you catch yourself thinking that it would be nice not to have to get up again tomorrow.

Climate change-related aspects

You bring a topic to today's lesson. Last week you talked to your partner about possible vacation destinations. Your partner expressed the wish to fly to the Caribbean for two weeks. She is convinced that you need a break from everyday life and also feels the need to lie on the beach for a while and do nothing. Financially, this is not a problem for either of you. However, you were at home just last weekend and had a long chat with your sister, who lives very sustainably, doesn't have a car out of conviction, buys local and vegetarian food and is a member of the German Nature and Biodiversity Conservation Union. In this conversation, your sister also brought up the subject of the extent to which

it is justifiable to continue living the way we do when you think about the children she teaches. You yourself follow the news relatively closely and are also of the opinion that far too little is being done in Germany to mitigate climate change. At the same time, you are aware that you do not have a sustainable lifestyle. When you suggested to your partner last week that you might choose a vacation destination that you can reach by car, she snorted, pointed out that it really wouldn't make any difference and then insinuated that you simply can't take two weeks off work and are therefore using concerns about the climate as an excuse.

Since then, the subject has stayed with you. You've read up on it again, and now you feel guilty every time you get in your car to drive to work. It seems downright absurd to you that we all produce so much more greenhouse gas every day than the planet can sustain - and even more absurd that you continue to do just that. You were on the Greenpeace website yesterday and had almost signed up to make a regular donation, but then you wondered if it would make any difference at all and closed the browser again. Your thoughts now often revolve around how hopeless the situation is. Your partner has already asked why you're in such a bad mood, but after the argument about the vacation, you don't want to tell her because you don't feel like you're being taken seriously. You feel helpless and stuck: continuing to live as you have up to now doesn't seem right; at the same time, it doesn't really matter whether you live sustainably or not, it won't stop climate change. But then you remember that you actually want children; is it even justifiable to have children when you know what their future will look like? On the other hand, mankind has found a solution for everything so far. But isn't that just blind optimism?

Course of therapy (6 hours so far)

You are generally quite open to psychotherapy, but you don't tell anyone other than your partner that you are taking advantage of it. You have had 6 sessions so far and have agreed with your therapist to initially apply for a short-term therapy of 12 sessions to check together whether psychotherapy could be helpful for you. You like your therapist and find her to be very understanding. You also notice that it is good for you to talk about the things that are bothering you: At work, you have a new supervisor who makes demands that cannot be met, which you then have to pass on to your coworkers; your partner actually wants to try to have a child now, but you would like to wait a year to try as agreed; and finally, your father was diagnosed with cancer a year ago and it is foreseeable that he will pass away in the near future. You can't really put your finger on what makes you feel good, the feeling is very vague and intangible for you, which also annoys you. So far in therapy, you have mainly talked about all these things. The therapist has repeatedly tried to work with you to establish a connection to your experience of pain. It seems plausible to you that you are under constant tension and that this is where the pain comes from; however, you cannot really feel this tension and also see no real possibility of how you could change something in your everyday life in order to be less tense.

Start of lesson

You have decided to discuss your thoughts on climate change and the argument with your girlfriend about your vacation destination in therapy today. Although this is not directly related to your reasons for starting psychotherapy, you hope that it will give you more clarity and peace of mind. You start the session by describing the situation:

"Two weeks ago, my partner and I talked about our choice of vacation destination. I had already told her that Miriam would like to go away for longer. Well, she actually wants me to go away for longer... anyway, I was visiting my parents at the weekend and saw my sister. We talked about the amendment to the Building Energy Act and my sister was very much in favor of installing a heat pump in my parents' house. I then read up on the subject, as my professional opinion was needed. And well... I also read a lot about climate change and our emissions. And that got me thinking a bit. I then asked Miriam if we wanted to go to the North Sea so that we didn't have to fly. Miriam didn't like that at all. Of course, we made up before we went to bed, that's our rule, but we haven't decided where we're going yet, and that's what's bothering me."

Please remain silent afterwards and wait for the therapist's reaction...

Case history (III) seminar on psychological stress caused by the climate crisis

Mrs. Dobrika, 49 years old

Mrs. D., married, resident in Dielheim, 3 children, nurse.

Complaints

You feel severely depressed and listless. You have been in this state ever since you started thinking intensively about the effects of climate change. You believe that it is long too late to save the planet. Sometimes you lie awake for hours at night because you are so ashamed of doing all this to the next generation. Meanwhile, you feel empty most of the time. You have no strength for anger or rage, and your grief no longer shakes you. If you were to die of a heart attack tomorrow, it would be a relief. However, you would never actively hurt yourself or try to kill yourself, you could never do that to your children. However, you no longer have much interest in leaving the house and going out into nature, which only makes you sadder. At first you continued to go to work, but two weeks ago you had your GP put you on sick leave because you were unable to concentrate and overworked at work and simply couldn't bring yourself to get up on time one morning. At the moment, you are still looking after your children and the household, but you feel guilty because you are paying much less attention to your children than you used to. You spend most of your time lying in bed or on the sofa knitting, you don't consume any media, you can hardly stand it: if there is no talk about climate change, you ask yourself how anything else can be considered relevant. When climate change is discussed, you become even more desperate because all the facts are there and yet nothing is being done. You had a phase after the birth of your first child in which you were paralyzed and hopeless for a while and perceived life as meaningless; you associated this with your new life situation and forced yourself to be active anyway, and after a few months you were fine again. Now, however, you see no possibility of changing your condition on your own.

Current triggers

On your last visit to Tyrol two months ago, you walked a route that you enjoyed walking as a child. You passed a place where you used to have a good view of a glacier. When you passed it this time, it had almost disappeared and the images of then and now were superimposed in your mind's eye. Since then, a leaden heaviness has stayed with you. Whereas you used to love going into the forest with your children at the weekend, you now feel a lump in your throat every time you look up at the treetops. When your children are enchanted by the sight of a squirrel running up the tree, tears well up in your eyes. You were also aware before that climate change was threatening the ecosystem, but this thought did not affect you in your daily life. Now you feel paralyzed, hopeless and cry when you think about all the things your children will no longer be able to experience and what a changed world they will have

to grow up in. Your husband was very understanding at first, but when you went on sick leave he made you promise to seek psychological counseling.

Life story

Family of origin

They come from a village in Tyrol, where they also went to school and graduated from high school. Your father works in agriculture, your mother is a housewife and has always worked in various jobs, as a seasonal worker in agriculture, as temporary help in the canteen of a large company and as a seamstress. You have four siblings who are all younger than you (f, -2; m, -5; f, -7). Your youngest sister now lives in the family home and also works in agriculture. Her father died of a heart attack a few years ago and her mother has been in an old people's home

with Alzheimer's for 2 years. The youngest sister had previously cared for her at home and has now taken over the house; there was no dispute about this between the siblings, nobody wanted to stay in the village except her. The older sister graduated from secondary school and trained as an industrial clerk and works and lives in the next largest city, her brother studied medicine and works as a radiologist in a hospital in Vienna. The relationship with all the siblings is very cordial, although you have the closest relationship with your brother in Vienna and talk on the phone about every two weeks. You visit your sister at home regularly, you have little contact with your sister in the neighboring town, but you are always happy to see her. The middle siblings are married and have 2 children each, her youngest sister was with her childhood friend until she was 33, after the separation she moved back in with her parents and has not been in a steady relationship since. She has a son from the relationship who she is bringing up on her own, she only has sporadic contact with the father, the son visits him every 14 days over the weekend.

Partnerships

You were together with a boy from your class from 15 to 18, the relationship ended because you moved to Vienna and he stayed in your home village. In Vienna you had different partners, but never for longer than half a year. That was a very exciting time for you and you have fond memories of it. You finally met your current husband in a bar when you were 25. He is 5 years older and was working as a lawyer for his company in Vienna at the time. A very passionate relationship quickly developed between the two of them. At the time, you worked shifts at the hospital, and for a year you took on as many weekend and night shifts as possible; this meant that you always had periods of one or two weeks without a shift, during which you would travel to your partner in Mannheim. After a year, you finally moved in with him. You married at the age of 29 and had three children (female, 18; male, 15; female, 13). It took two years before you got pregnant for the first time, so you are very grateful that it worked three times in

total. Basically, you would have liked to have a fourth child, but again you didn't get pregnant and then discussed with your partner at the age of 44 to stop trying because you felt too old to become a mother again.

Work / Professional

After leaving school, you also went to Vienna and trained as a nurse. You lived there until you were 26 and enjoyed it very much. Then you and your current husband moved to Germany, first to a city apartment in Mannheim and later to a house in Dielheim. You continued to work in oncology at the hospital in Mannheim at the beginning. After the birth of your first child, you stopped working and started again part-time three years ago when your youngest daughter started school. Now you work in day care, as the shift schedules here are more compatible with your responsibilities as a mother. You like your job and your colleagues, but you sometimes have conflicts with your boss because, from your perspective, she focuses more on profitability and less on the well-being of the patients.

Social situation / hobbies

At the beginning of your time in Germany, you lived with your partner in his city apartment in Mannheim. It was practical and affordable, but you didn't feel comfortable there. Your husband earns very well, so you were very grateful when you were able to move into a house in Dielheim after three years, which is also yours and has enough space for all the children. You feel at home here and you can get out into nature much more quickly. Your husband now works in Karlsruhe, which is easy to reach by car.

You have always been a very active person with lots of friends. You also played basketball at school, but you stopped when you moved to Vienna. But you still like to go jogging and attend a yoga class. You no longer do either at the moment due to your mood. Your current circle of friends consists largely of parents of friends of your children; your closest friend is the mother of a friend of your eldest daughter. She is a geriatric nurse and is very worried about you. They are regularly active in parents' councils and similar committees and are very well known in Dielheim. That's why you're worried that people are starting to talk because you haven't been to any events for weeks and have stopped going to work.

Psychological findings

You are dressed practically, but very colorless. They find it difficult to maintain eye contact and speak in a low voice at a normal speed. You are very worried about the future of your children on this planet, you are not worried about yourself. Your mood is basically desperate, but most of the time you just feel empty. Crying or getting upset takes too much energy. You wake up frequently during the night and then can't fall asleep because of circling thoughts. You still eat as usual, but you no longer enjoy it. Your

thoughts are tired of life, but you would never harm yourself because of your children. You usually feel tired and have the feeling that you are moving more slowly and with less energy than a few months ago.

Climate change-related aspects

At the beginning, you read a lot about climate change and its effects, watched reports on the subject and followed the profiles and activities of well-known climate activists like Luisa Neubauer on social media. However, you don't feel the need to get involved in activism, you don't believe that this could change anything. So you currently have a lot of specialist knowledge about the physical principles of climate change, but less about the political aspects.

Course of therapy (initial consultation)

You are ambivalent about psychotherapy. After all, their mood is a result of the fact that everything is hopeless and that we as a society have caused climate change and not stopped it. A psychotherapist can't change that, but you hope that you might be able to learn a different way of dealing with your feelings and thoughts. You are also torn by the idea that your children might be disappointed in you in a few years' time if you withdraw completely now. This thought finally motivated you to contact the psychosomatic outpatient clinic. You had an initial consultation with a doctor there, who was very friendly and recommended outpatient psychotherapy at the end. You are now back here for your first consultation with the therapist who was assigned to you.

Start of lesson

You assume that the first session will be about describing your symptoms so that the therapist gets an impression of you. So you start the session as follows:

"Thank you for making it today. As you've probably read in the doctor's letter, I'm feeling pretty bad."

Please remain silent afterwards and wait for the therapist's reaction...
